# Medical student Interest in expanded transgender gynecologic education

**DOI:** 10.12688/mep.18990.1

**Published:** 2022-02-08

**Authors:** Danielle Wright, Alexandra Campedelli

**Affiliations:** 1 Gynecologic Surgery and Obstetrics, Uniformed Services University of the Health Sciences, Bethesda, MD, 20814, USA

**Keywords:** Transhealth, Medical Education, Gynecology, Inclusivity

## Abstract

**Background: **The purpose of this study was to use survey data to better understand medical students’ comfort in taking care of transgender patients and to determine whether this is an area that needs to be expanded upon in their curriculum.

**Methods *:*** Eight pre-clerkship medical students participated in a virtual two-week course about gynecologic transgender care which included a mix of self-paced learning combined with two days of interactive faculty-led sessions. Students were asked to complete a pre and post course survey evaluating their comfort in caring for transgender individuals.

**Results** **:** Students’ knowledge about the gynecologic needs of transgender individuals significantly improved after taking the course with the average student rating before and after the course being 2.38 ± 0.74 (p<0.05) and 4.25 ± 0.46 (p<0.05), respectively. In addition, 100% of students “agreed” or “strongly agreed” that this course built their confidence in taking care of transgender patients in the clinical setting.

**Conclusions** **: **This study highlights a potential gap in medical education while also emphasizing that knowledge on this special population can enhance physician confidence when caring for transgender individuals.

## Introduction

In the United States, there are an estimated 1.4 million people who identify as transgender ^1^. Transgender individuals experience an increased prevalence of workplace discrimination, sexual and physical violence, poverty, HIV/AIDS, sex-work, lack of health care access, murder, depression, and anxiety ^2, 3^. Despite the adversary societal pressures and the toll each of the previously mentioned experiences can have on their overall health, transgender patients are much less likely to seek help in healthcare settings ^4^. According to participants in the National Transgender Discrimination Survey, 28% of transgender patients were less likely to seek healthcare to avoid discrimination and 50% of participants reported being treated by a provider who lacked knowledge on healthcare issues pertaining to transgender individuals ^4^. To create a welcoming, safe clinical environment for transgender and gender nonconforming patients, medical professionals should not only display visible and explicit statements of inclusivity but also use proper terminology and know their unique health care needs ^5^. Proper training of providers can lead to increased patient comfortability, improved patient-physician communication, and better healthcare outcomes ^6^.

Across the country, there is a gap in transgender medical education which can result in a relative discomfort in students, and ultimately physicians, in caring for transgender individuals ^7^. To provide equitable care, physicians should have an understanding of the specific health care needs of the population they’re serving and they should be able to tailor the care provided to the unique needs of the individual. For example, specific gynecologic needs for transgender men include: unchanged preventative health screenings depending on the presence of reproductive organs, hormone therapy, fertility preservation, and continuing annual screening for sexually transmitted infections depending on sexual practices ^8, 9^. The purpose of this study was to use survey data to better understand medical students’ comfort in taking care of transgender patients and to determine if this is an area that needs to be expanded upon in their curriculum. We hypothesized that students would feel increased comfort in caring for transgender patients after teaching facts about this specific population.

## Methods

### Study design

This study was approved by the Institutional Review Board (IRB) of the Uniformed Services University of the Health Sciences and Walter Reed National Military Medical Center (approval number: DBS.2021.263). From January to August 2021, second- and third-year medical students were able to register for a two- week virtual course on gynecologic care for transgender individuals. A total of eight students that participated in the course. The class was designed such that from Monday to Thursday students participated in self-paced learning for four to five hours per day. Educational materials included publications from the American College of Obstetricians and Gynecologists (ACOG), University of California at San Francisco’s (UCSF) Transgender Care & Treatment Guidelines, online videos, and articles ^8, 10– 14^. Week 1 content focused on an overview of transgender medicine with topics including: creating a safe clinical environment, disparities, gender affirming therapy & surgery, contraception, and screening for sexually transmitted infections. Week 2 focused on an overview of gynecological care for transgender individuals including: cervical and prostate cancer screening, breast cancer screening, pelvic pain, abnormal uterine bleeding, and fertility preservation. On Fridays they met with their faculty preceptor to review the material, answer questions, and simulate patient counseling.

### Consent

Students were asked to complete a pre and post course survey evaluating their comfort in caring for transgender individuals using Google Forms; the survey was optional, and each participant was informed of this fact. Written informed consent was waived after the IRB considered our study’s nature and use of anonymous data. Oral consent to partake in the survey was obtained by each participant and documentation of consent was made by way of their participation in the survey.

### Analysis

Questions were posed to students used a scale from “Strongly Disagree” to “Strongly Agree.” These results were then converted to a five-point scale for analysis. In addition, students were able to provide feedback in an open-ended fashion. The data was analyzed using a Student's two sample t-test assuming equal variance; significance was determined at a p-value less than 0.05. Our data can be reviewed on Figshare ( *Underlying data*) ^15^.

## Results

### Virtual experience

On the pre-course survey, 100% of students “agreed” or “strongly agreed” that they felt comfortable with computers and technology. They were “neutral,” “agreed,” or “strongly agreed” that taking the course virtually would have a positive impact on their educational experience. On the post-course survey, 100% of students “strongly agreed” that the virtual meetings expanded their knowledge and faculty were available if they had questions or concerns.

### Course content

On the pre-course survey, 100% of students “agreed” or “strongly agreed” that the learning objectives were clear, the course content was organized and well planned, and that the course workload seemed appropriate. On the post-course survey, 100% of students “agreed” or “strongly agreed” that completing the assignments reinforced the material learned during the course. Recommended content for future courses included: an in depth review of gender reassignment surgery, a panel discussion with transgender patients, and providing more specific facts about transgender care in the military including Tricare coverage for specific services pertaining to transgender individuals.

### Student knowledge

**Figure 1.**
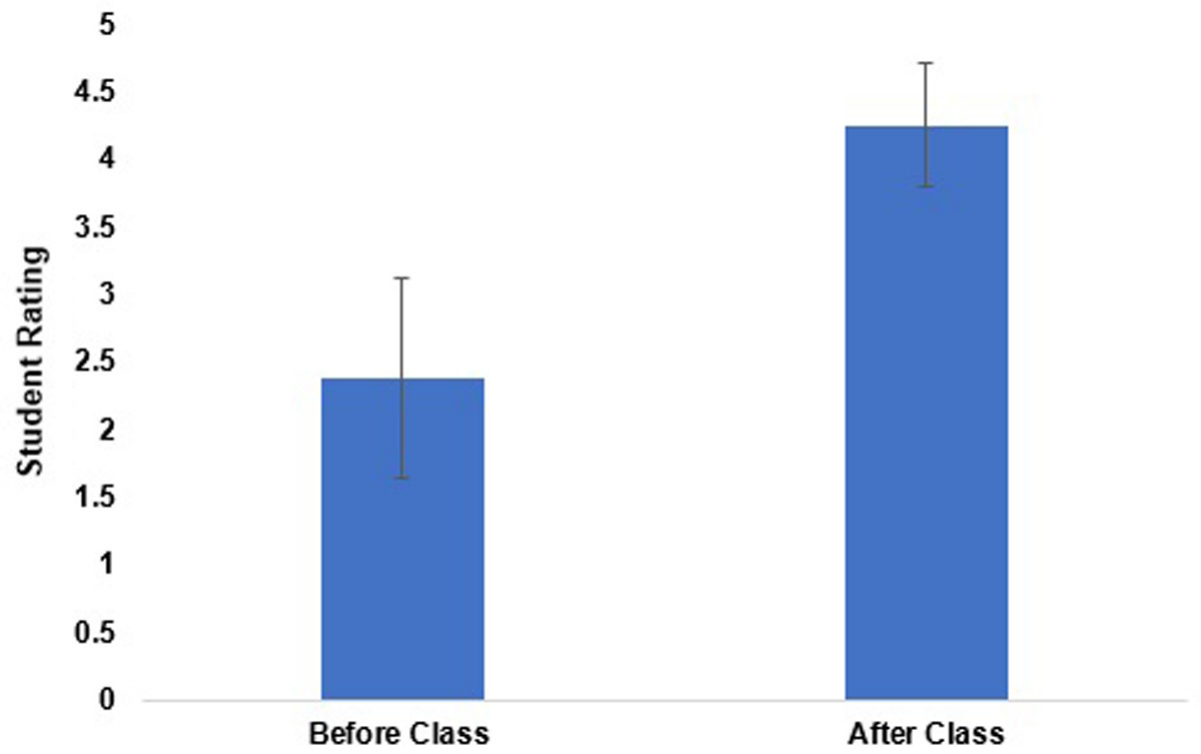
Comparing student self-ratings of their skill in caring for transgender patients in the clinical setting before and after taking the course. The mean skill level was 2.38 ± 0.74 before the course and 4.25 ± 0.46 after the course. The difference between the two groups was statistically significant (p<0.05).

On the pre-course survey, students were asked to rate their level of knowledge on the gynecologic needs of transgender individuals. The average rating at the start of the course was 2.38 ± 0.74 points. On the post survey the students were asked to rate their knowledge on this topic; the average student rating was 4.25 ± 0.46 points ( Figure 1). The difference between the pre and post-course survey was statistically significant.

### Future impact

**Figure 2.**
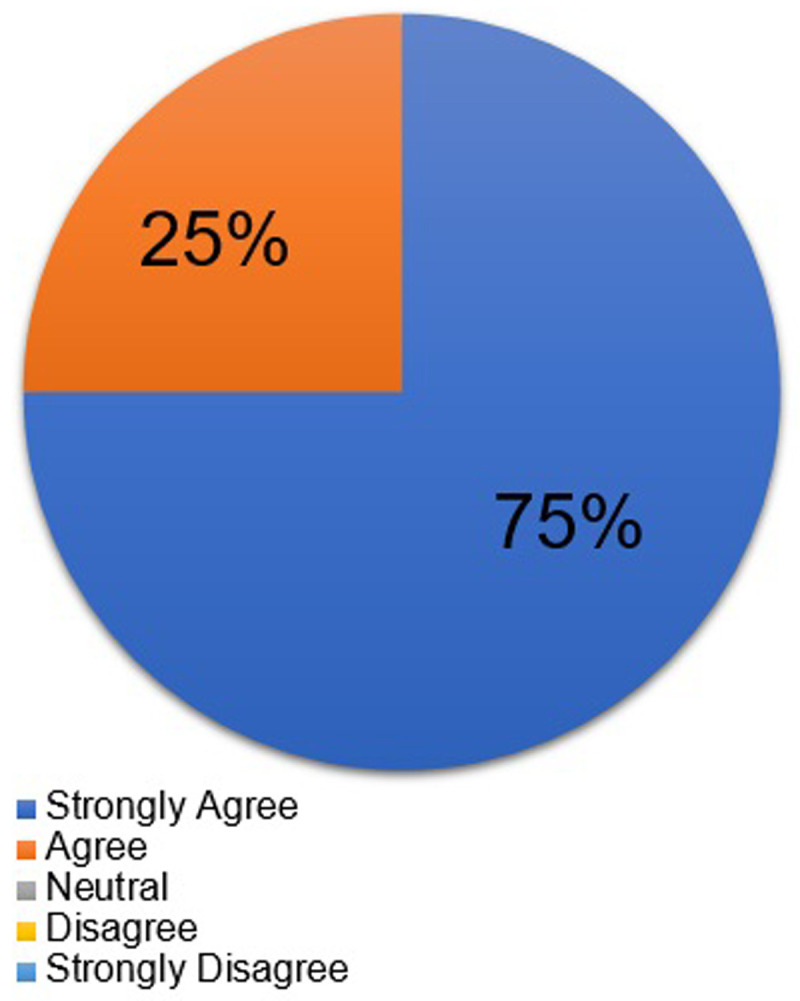
Students' rating on the statement that “this course built my confidence in taking care of transgender patients” after course completion.

In regards to the students’ view on the significance of transgender education, 100% of students “strongly agreed” that the content of the course was important. On the pre-course survey, Table 1 shows the students’ responses as to why they chose the course. By the post-course survey, 100% of students “agreed” or “strongly agreed” that this course built their confidence in taking care of transgender patients in the clinical setting ( Figure 2), and 87.5% of students stated that they would recommend this course to other students.

**Table 1.** Student Pre-Survey Responses to “Why did you choose this course?”.

“I am very interested in a career in OBGYN and I know that learning about transgender medicine is extremely important in order to provide care to ALL patients…”
“I have always been interested in LGBT health, and really appreciate the opportunity to become conversant (maybe not fluent) in trans healthcare. Largely, it is an opportunity to begin to right some of the wrongs done to various gender and sexuality minority groups by the medical establishment”
“I don't know much about gynecologic care for trans patients, and I want to be able to take care of my future patients well. I also think this population is vulnerable and physicians at large may not have the knowledge/tools to take care of this demographic; I want to be a physician that can and that can pass that knowledge to my peers.”
“I’m Interested in transgender care in the military and I want to be knowledgeable for all my future patients so I can best serve them…”
“I want to be inclusive in my future practice, no matter the specialty I go into! I am hoping this course will give me the knowledge and skills necessary to create a safe and inclusive space for my patients.”
“I am interested in gynecology and I have not yet worked with this population. Our exposure so far has been limited and I was interested in learning more.”
“…This topic is so relevant to the changing populations and care we provide today!”

## Discussion

This study highlights a gap in transgender medical education and emphasizes that building students’ knowledge in transgender topics can enhance their comfort in taking care of this special population. Overall, we saw a significant improvement in the self-rated knowledge scores of students in taking care of transgender individuals in the clinical setting after the course. Themes surfaced on why the students chose to take this course include: (1) a recognition of the injustices in the healthcare system among transgender patients, (2) an interest in expanding their knowledge, and (3) providing a safe and inclusive environment for their patients.

### Barriers to creating transgender curricula in undergraduate medical education

Multiple studies have highlighted the need for transgender medical education ^16– 18^. In 2011, 33% of medical schools reported that they did not teach specifically on topics related to individuals in the lesbian, gay, bisexual, transgender, and queer (LGBTQ) community ^16^. For the schools that did provide this learning content, the specific training averaged five hours of care over students’ four-year curriculum ^16^. In order to reduce health care disparities and to effectively care for members in the LGBTQ community, wide implementation and standardization of this type of medical education should be applied ^19^. Challenges to adopting transgender education into curricula include: ignorance towards knowing the need exists, time limitations when building a curriculum, and lack of institutional support ^20^. However, Tamas and colleagues did show that barriers such as instructor perceived lack of relevance can be largely be mitigated with increased faculty development ^21^.

### Transgender medical education can lead to better patient care

In 2017, surveyed internal medicine residents did not feel comfortable providing care to transgender patients, with 55% of respondents stating they did not receive transgender-specific health care training in medical school ^22^. In a study among gynecologists, despite the comfort 92.9% of those surveyed had caring for LGBTQ patients, only 25.3% were comfortable caring for transwomen and 29% caring for transmen. Among gynecological physicians, 80% did not receive specific transgender care in their residency training ^23^. Supporting this fact, in 2016 only 36.9% of directors of gynecologic residency programs reported transgender patients being within their served population; this referenced study highlighted that training via online models and lectures such was those published by ACOG and UCSF could be a viable alternative to fill this void ^8, 10– 14, 24^.

 The Association of American Medical Colleges (AAMC) provide a framework for transgender medical education that promotes an inclusive culture and professional competency ^25^. The COVID-19 pandemic challenged this framework as medical schools pulled students from the hospital forcing them to adapt to virtual classrooms. However, easy access to a plethora of resources afford the opportunity for self-directed, online learning as seen with this course ^26, 27^. This study proved that a virtual platform is an effective way to learn transgender topics; further, student feedback highlighted their desire to take their virtual learning a step further through the implementation of case-based learning sessions where they can practice and improve interview and patient trust-gaining skills. Focused, transgender medical education can change physicians’ attitudes towards caring for this population ^17, 28– 31^. Ultimately, gender-affirming providers can improve patient care through addressing the specific health needs of their patient ^15, 32– 35^.

### Limitations

With only eight participants, the results of this study may not be generalizable to the larger population as self-selection of the course could have introduced bias based on those who were interested in gynecologic or transgender medicine. Secondly, our study lacked a control group. Thirdly, follow up with participants once they are practicing medicine would be warranted in order to fully assess the benefit of this course.

## Conclusions

Physicians who are comfortable taking care of transgender patients are more likely to create safe, inclusive environments that foster a collaborative patient-physician relationship. Gynecologic care should be tailored to the unique health needs of transgender individuals in order to provide equitable health care. Increasing medical education about the unique needs of the transgender population is one step closer to resolving the inequalities that transgender individuals face in our health care system.

## Data availability

### Underlying data

Figshare: Student interest transgender gynecologic education, https://doi.org/10.6084/m9.figshare.18866441.v2 ^15^

This project contains the following underlying data:

- TG Pre-Course Survey.csv
- TG Post-Course Survey.csv

### Extended data

Figshare: Student interest transgender gynecologic education, https://doi.org/10.6084/m9.figshare.18866441.v2 ^15^

This project contains the following extended data:

- Fig1and2TransMedOBGYN_22.xlsx (scores dataset)

Data are available under the terms of the Creative Commons Zero "No rights reserved" data waiver (CC0 1.0 Public domain dedication).
