# Supplementary material for: Pilot Study: Increasing Medical Student Comfort in Transgender Gynecology
Source: MedEdPublish (2016). 2022 Jul 11;12:8. Originally published 2022 Feb 8. [Version 2] doi: 10.12688/mep.18990.2 (PMC9370083; doi:10.12688/mep.18990.2)
Supplement: Peer review history

# Peer review: Reviewer response for version 2

**DOI:** 10.21956/mep.20590.r32296

**Published:** 2022-07-18

**Authors:** Talia Stewart

**Affiliations:** 1 MetroHealth Medical Center, Cleveland, OH, USA

Thank you to the authors for adjusting the title and including this is a pilot study.

Have any limitations of the research been acknowledged?

Partly

Is the study design appropriate and does the work have academic merit?

Partly

Is the work clearly and accurately presented and does it cite the current literature?

Yes

If applicable, is the statistical analysis and its interpretation appropriate?

Yes

Are all the source data underlying the results available to ensure full reproducibility?

Yes

Are the conclusions drawn adequately supported by the results?

Partly

Are sufficient details of methods and analysis provided to allow replication by others?

Yes

Reviewer Expertise:

Transgender access to gyn care in rural New Hampshire (chart review of over 200 patients)

I confirm that I have read this submission and believe that I have an appropriate level of expertise to confirm that it is of an acceptable scientific standard.

# Peer review: Reviewer response for version 1

**DOI:** 10.21956/mep.20347.r32172

**Published:** 2022-06-29

**Authors:** Talia Stewart

**Affiliations:** 1 MetroHealth Medical Center, Cleveland, OH, USA

The purpose of this study is to survey medical students to determine whether or not gynecological transgender health should be expanded upon in their medical school curriculum. The authors found a significant improvement in students' comfort in taking care of transgender patients after the school implemented a two-week course. These findings are not surprising, in my opinion, due to the fact that so little is known by providers about taking care of a transgender patient that any education on this topic will increase comfort with taking care of this population. Thus, I think it is commendable that this medical school is prioritizing this and that this study has been done. 

 I think two things make the significant study results hard to interpret/overinflated. For one, the sample size is low. Also because participants self-selected, they were likely to gain much from this course and so the significant results are likely overinflated. I think it will be helpful to address things that will likely need to be fixed with the course moving forward to make sure that students who may be biased against the LGBTQ population also gain from the course--for example administering exams. 

 Unfortunately, I would not accept this for passing peer review. I think that due to the low sample size and selection bias, the results are not surprising and I do not think it gives the reader (ie medical school curriculum developers) a clear way on how to integrate this into the curriculum. I think the authors need a larger sample size or to implement this into the curriculum of one of their classes and then publish those results instead and it will then be a stronger manuscript.

 Lastly, please include a line about the percentage of survey response! I assume it is 100% but I do not see it mentioned here.

Have any limitations of the research been acknowledged?

Partly

Is the study design appropriate and does the work have academic merit?

Partly

Is the work clearly and accurately presented and does it cite the current literature?

Yes

If applicable, is the statistical analysis and its interpretation appropriate?

Yes

Are all the source data underlying the results available to ensure full reproducibility?

Yes

Are the conclusions drawn adequately supported by the results?

Partly

Are sufficient details of methods and analysis provided to allow replication by others?

Yes

Reviewer Expertise:

Transgender access to gyn care in rural New Hampshire (chart review of over 200 patients)

I confirm that I have read this submission and believe that I have an appropriate level of expertise to state that I do not consider it to be of an acceptable scientific standard, for reasons outlined above.

# Response

**Published:** 2022-06-30

**Authors:** Danielle Wright

**Affiliations:** Uniformed Services University of the Health Sciences, USA

We thank Reviewer 2 for taking the time to read and review this paper.  Reviewer 2 highlighted that this paper would not pass peer review because of the items listed in our limitations section.  Unfortunately, increasing our sample size isn't possible at this time because the course isn't apart of the curriculum and was provided as an elective during the height of the pandemic when medical education shifted to virtual.  Thus, now throughout the paper and in the title this study is being addressed as a pilot study to reflect that we are trying to prove a concept and highlight the value of this curriculum.  Lastly, we did add several lines to address our response rate.

# Peer review: Reviewer response for version 1

**DOI:** 10.21956/mep.20347.r31591

**Published:** 2022-03-21

**Authors:** Lindsey Choi

**Affiliations:** 1 Blanchfield Army Community Hospital, Fort Campbell, KY, USA

The authors use surveys to confirm a gap in the medical education training for transgender individuals in the United States while offering a pathway for improvement in this educational gap. The introduction elucidates the difficulties that transgender men and women may face while exploring the difficulties specifically within the field of medicine. The methods section was clear and the study design is easy to replicate for future studies with the surveys available in the underlying/extended data sections. The results were presented in a manner that is easy to understand. The discussion addressed barriers to the current curriculum in medical education while providing solutions provided through online resources from major medical organizations as used for this study. The authors also recognize limitations to their study, including small participant size and the application of these study results to the larger population. Overall, the authors were successful in summarizing a gap in the current medical training and were able to determine that exposure to the information through online resources may improve inequalities of transgender medical care by training medical students earlier in their careers. 

 While addressed in the "Analysis" subsection of "Methods", the data in "Student knowledge" subsection of "Results" may be better represented if the definitions of the numbers are reintroduced in this section or if that entire subsection was incorporated into the results.

Have any limitations of the research been acknowledged?

Yes

Is the study design appropriate and does the work have academic merit?

Yes

Is the work clearly and accurately presented and does it cite the current literature?

Yes

If applicable, is the statistical analysis and its interpretation appropriate?

Yes

Are all the source data underlying the results available to ensure full reproducibility?

Yes

Are the conclusions drawn adequately supported by the results?

Yes

Are sufficient details of methods and analysis provided to allow replication by others?

Yes

Reviewer Expertise:

Obstetrics, Gynecology, Infertility

I confirm that I have read this submission and believe that I have an appropriate level of expertise to confirm that it is of an acceptable scientific standard.
